# Ultrasound-guided transmuscular quadratus lumborum block reduced postoperative opioids consumptions in patients after laparoscopic hepatectomy: a three-arm randomized controlled trial

**DOI:** 10.1186/s12871-021-01255-3

**Published:** 2021-02-11

**Authors:** Mengya Pang, Guoliang Sun, Weifeng Yao, Shaoli Zhou, Ning Shen, Haofeng Liao, Hanbing Xie, Wanling Gao, Mian Ge

**Affiliations:** Department of Anesthesiology, The Third Affiliated Hospital of Sun Yat-sen University, 600 Tianhe Road, Guangzhou City, 510630 Guangdong Province China

**Keywords:** Postoperative analgesia;laparoscopic hepatectomy, Transmuscular quadratus lumborum block;oxycodone

## Abstract

**Background:**

To investigate whether transmuscular quadratus lumborum block (TQLB) combined with oxycodone-based patient-controlled intravenous analgesia (PCIA) compared with sufentanil-based patient-controlled intravenous analgesia could reduce postoperative pain and opioid consumption in patients undergoing laparoscopic hepatectomy.

**Methods:**

Eighty patients undergoing laparoscopic hepatectomy surgery were randomly divided into Group S (Sufentanil for PCIA group), Group O (Oxycodone for PCIA group) and Group QO (transmuscular quadratus lumborum block + oxycodone for PCIA group). Primary outcome was Numerical Rating Scale (NRS) pain score when coughing at 6th hour after the operation. We summarized opioid consumption and recorded complications, opioid drug adverse reaction and analgesia satisfaction.

**Results:**

NRS pain scores were significantly lower in Group QO while patients coughing at 6th hour after the operation compared with Group S and Group O (median (interquartile range [IQR]):Group S vs. Group O vs. Group QO 4.0 [3.0, 5.0] vs. 4.0[3.0,5.0]vs.3.0 [2.0, 3.0], *p* < 0.05). Within 24 h after surgery, the bolus times of PCIA (patient controlled intravenous analgesia) in the QO group was reduced which was compared with the Group S and Group O (median (interquartile range [IQR]):Group S vs. Group O vs. Group QO 13.0 [10.3, 19.5] vs. 11.5 [7.8, 18.3]vs.6.5[3.5,12.0], *p* < 0.05). The proportion of patients in the three groups who required additional analgesia was ranked as Group QO < Group O < Group S(*p* < 0.05). The analgesic satisfaction of patients in Group QO was higher than the Group S (*p* = 0.001) and Group O (*p* = 0.012).

**Conclusions:**

TQLB combined with oxycodone-based PCIA provided satisfactory postoperative analgesia and reduced oxycodone consumption in patients following laparoscopic hepatectomy.

**Trial registration:**

ChiCTR1900028467 (22/12/2019).

**Supplementary Information:**

The online version contains supplementary material available at 10.1186/s12871-021-01255-3.

## Introduction

Liver cancer is a common cause of cancer death, and 75–85% of liver cancer patients are diagnosed with hepatocellular carcinoma (HCC) [1]. Laparoscopic hepatectomy is a commonly performed surgical procedure for patients with HCC [2, 3]. Compared to open procedures, laparoscopic surgery may still produce severe pain in the early postoperative period. A study showed that a significant proportion (46%) of patients after abdominal surgery with severe postoperative pain were those who underwent laparoscopic surgery, and they reported more intense pain and required a larger dose of analgesics than the patients after laparotomy (54%) [4]. Opioids remain the cornerstone in postoperative pain management. Among different types of opioids, several studies showed that oxycodone provided better visceral pain relief than morphine [5, 6]. However, opioid-related adverse reactions, such as nausea, vomiting, pruritus, respiratory depression, and delayed intestinal function recovery may compromise postoperative recovery of patients undergoing surgery for cancer [7].

Enhanced recovery after surgery (ERAS) strategies aims to improve postoperative outcomes and has been increasingly applied in a variety of surgical procedures. Multimodal analgesia is an important part of ERAS, targeting multiple mechanisms and pathways by using different types of analgesics to optimize analgesic effects and minimize adverse reactions [8, 9]. Among these, regional nerve block is a widely used technique which plays a significant role in ERAS [10–12]. Transmuscular quadratus lumborum Block (TQLB) is a novel technique of abdominal wall nerve block, which has been shown to provide effective pain relief after upper and lower abdominal surgery [13–15]. To date, however, the effects of TQLB on postoperative pain in patients who receive patient-controlled intravenous analgesia (PCIA) with oxycodone after laparoscopic hepatectomy is still unclear.

In this study, we hypothesized that multimodal analgesia of TQLB combined with oxycodone-based PCIA compared with oxycodone-based PCIA or sufentanil-based patient-controlled intravenous analgesia could improve postoperative pain control, reduce opioid consumption, and patients could have better analgesic satisfaction.

## Methods

### Patients

This is a prospective, single-blind, randomized controlled trial. After the study protocol was approved by the medical ethics committee of the Third Affiliated Hospital of Sun Yat-sen university (No.[2018]02–427-01), this trial was registered at the Chinese Clinical Trial Register (http://www.chictr.org.cn, identifier: ChiCTR1900028467) on December 22, 2019. Written informed consent was obtained from each patient. 100 patients who scheduled for elective laparoscopic hepatectomy were enrolled for eligibility between November 11, 2018 and June 15, 2020.

**Fig. 1:**
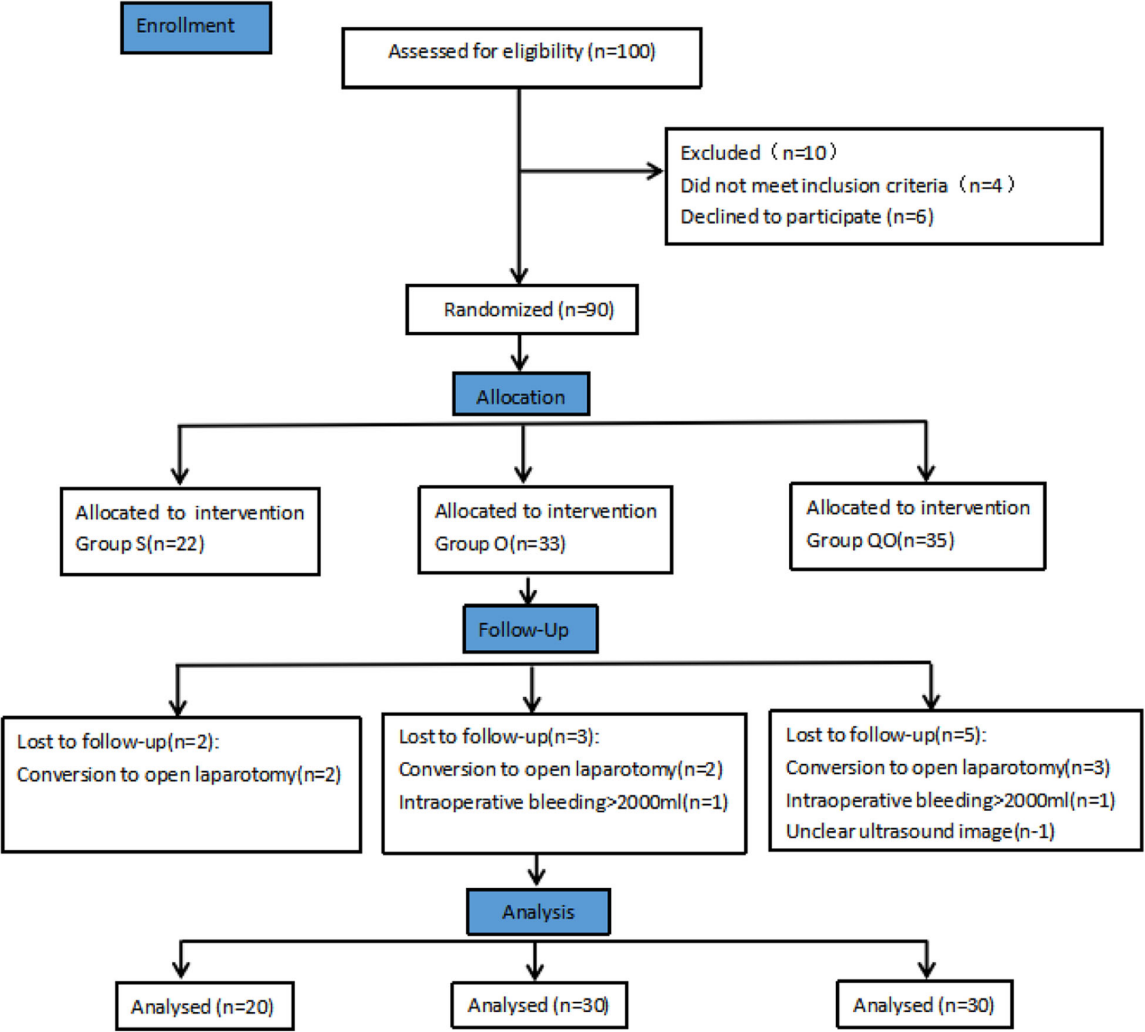
Consort diagram. S,sufentanil;O,oxycodone;QO,quadratus lumborum block and oxycodone

Inclusion criteria were American Society of Anesthesiologists (ASA) physical status I-III, 18–80 years old, 50–70 kg, and liver function of Child A and B grade. Patients with significant dysfunction of heart, brain, lung, liver, or kidney, Child C grade of liver function, immune system dysfunction (rheumatoid arthritis, multiple sclerosis, diabetes, thyroid disease), shift to open surgery, allergy to local anesthetics or any other medication used in this study, mental illness, infection at the puncture site, peripheral neuropathy, coagulation dysfunction, opioid abuse, inability to cooperate with Numerical Rating Scale (NRS) for pain or use of PCIA device, or serious adverse events occurred during the operation (such as serious cardiovascular events and anaphylactic shock) were excluded from the study. Eligible patients were randomly assigned into sufentanil for PCIA (Group S), oxycodone for PCIA (Group O) and transmuscular quadratus lumborum block with oxycodone for PCIA (Group QO) using a computer-generated randomization sequence and the allocation ratio was Group S:Group O:Group QO = 2:3:3. The allocation concealment was achieved by using sealed opaque envelopes. A trained observer who was blinded to groups assessed postoperative outcomes among patients. The postoperative observer, the researcher who analyzed the data, and patients were masked to the group allocation (Fig. 1) and patients were blinded after assignment to interventions.

### Anesthesia

All patients received standardized general anesthesia. For anesthesia induction, midazolam 0.04 mg/kg, propofol 1.5–2 mg/kg, and sufentanil 0.3 μmg/kg were administered. Cisatracurium 0.6 mg/kg was used to facilitate tracheal intubation. To maintain the anesthesia, sevoflurane in a mixture of O_2_/air was used and adjusted to Narco Trend index 36–56. Remifentanil was administered as needed to control the changes in heart rate and blood pressure within ±20% of the baseline values.

We set the equivalent dose ratio of sufentanil to oxycodone was 0.01:10 in this research. At skin closure,0.05μg/kg sufentanil and 5 mg tropisetron were given in Group S. Meanwhile 0.05 mg/kg oxycodone and 5 mg tropisetron were given at the time of suturing skin in Group O and Group QO. Each participant would receive standard postoperative intravenous patient-controlled analgesia (PCIA) at the end of operation. A 1μg/ml sufentanil-added saline solution was installed on automatic injection pump (Apon, China) in Group S and a 0.5 mg/ml oxycodone-added saline solution was installed on automatic injection pump (Apon, China) in Group O and Group QO. The automatic injection pump was adjusted to background dose of 0.5μg/h for sufentanil and 0.5 mg/h for oxycodone, bolus dose of 0.03 μg/kg for sufentanil and 0.03 mg/kg for oxycodone, lockout interval of 10 min and maximum dose of 10 ml/h.

### Ultrasound-guided TQLB

**Fig. 2:**
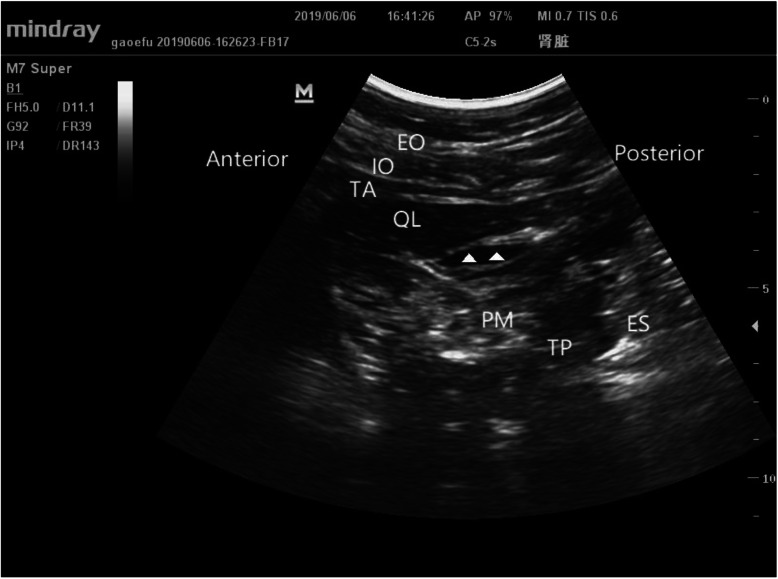
The Shamrock sign. Sonographic model image of transmuscular quadratus lumborum (TQL) block. Anatomy relevant for the execution of the TQL block. The needle is inserted in plane to the curvilinear probe and advanced through the quadratus lumborum (QL) muscle to reach the interfascial plane(▲, local anesthetics) between the QL and psoas major (PM) muscles. Injectate spreads in the interfascial plane and depresses the PM muscle. TP, transverse process; PM, psoas major; QL, quadratus lumborum muscle; ES, erector spinae; EO, external oblique muscle; IO, internal oblique muscle; TA, transversus abdominis muscle

Immediately after the surgery, bilateral transmuscular quadratus lumborum block was performed under the ultrasound guidance in the Group QO. Patients were in a supine position under general anesthesia. A thin pillow was placed under the hip to gain a better ultrasound image. For ultrasound scanning, a curved probe (Mindray Ultrasound Scanner, M7 Super diagnostic ultrasound system, Mindray, China) was placed vertically to the iliac crest at the posterior axillary line to identify the Shamrock sign. A 21-G needle (100-mm needle for peripheral nerve blocks, Hakko, Japan) was then inserted by using an in plane technique, and the top of the needle was directed to the quadratus lumborum (QL) muscle under the guidance of ultrasound. Once a proper location of the needle tip between the psoas major muscle and the QL muscle was confirmed, 30 ml of 0.25% ropivacaine was injected into the interfascial plane on each side. A successful blockade was indicated by the diffusion of local anesthetics around the QL muscle under the ultrasound (Fig. 2). All blockade procedures were performed by an experienced anesthesiologist. For the Group S and Group O, TQLB was not performed.

### Primary outcome and pain assessment

The predefined primary outcome of this study was the NRS pain score while coughing at 6 h after surgery. In addition, NRS pain scores at postoperative 2, 6, 12, 24, 48, 72 h were also recorded and analyzed.

In the NRS (0 to 10), 0 indicated no pain, and 10 indicated the most intense pain. Taking the procedures of laparoscopic hepatectomy into account, pain assessment was performed in four domains: pain when coughing, pain at rest, incision pain and visceral pain. Specific questions were used respectively: “What is NRS pain scores when you cough”, “What is your NRS pain scores when you lay down in a quiet situation”, “What is your NRS pain scores on the incision site” and “What is your NRS pain scores about dull pain inside the abdomen”.

### Other outcomes

We recorded the number of bolus of PCIA and oxycodone consumption at 0–2,2-6,12–24, 24–48, and 48-72 h after surgery and postoperative self-controlled usage of sufentanil or oxycodone. Postoperative sedation level was assessed by using the Rammsay sedation scale (1 = anxious, agitated, or restless; 2 = cooperative, oriented, and tranquil; 3 = responds to command; 4 = brisk response to a light glabellar tap or loud auditory stimulus; 5 = sluggish response to a light glabellar tap or loud auditory stimulus; and 6 = no response to the stimuli).

We also recorded parameters of vital signs and postoperative recovery such as numbers of bowel sounds at 2, 6, 24, 48 and 72 h after surgery, time of first exhaust, intaking and going to ground and time of removing the urine tube were recorded either and patients’ satisfaction to analgesia. Patients were asked to rank their satisfaction according to the following scale: 1 = very unsatisfactory; 2 = unsatisfactory; 3 = neutral; 4 = satisfactory; 5 = very satisfactory.

### Side effects and adverse events

Side effects including nausea and vomiting were recorded at 2, 6, 24, 48 and 72 h after surgery. We monitored adverse events including respiratory depression, local anesthetic toxicity, lower limb paralysis, hematoma, and organ injury. In case of these events, immediate treatments could be taken accordingly.

### Statistical analysis and sample size calculation

The primary outcome of the study was NRS scores when coughing at 6th after the operation. Our preliminary analysis determined that the NRS scores when coughing were 4.0 ± 1.0vs3.0 ± 0.5vs2.2 ± 0.8 in Group S vs Group Q vs Group QO. The sample size was calculated by the PASS version 15.0.5(NCSS, Kaysville, Utah, USA). The grouping ratio of 2:3:3 and the follow-up rate were calculated for 20 patients in the Group S and 30 patients in the Group O or Group QO respectively (considering the 20% loss of follow-up rate), and the efficacy of 0.80 was obtained to detect the significant difference between the mean values of the two groups (bilateral mean = 0.05).

Variables were presented as mean (SEM), median (IQR), count (%), and range as appropriate. Data for NRS scores, the number of bolus times in PCIA, opioids consumption and vital signs were analyzed by ANOVA for repeated measures. Other data for abnormal distribution or rank variable were analyzed by the Kruskal-Wallis test for inter-group comparisons and Bonferroni method for pairwise comparison. Qualitative variables were analyzed by the Chi-squared test or Fisher’s exact test. SPSS version 20.0 (IBM Corporation, Armonk, NY, USA) and GraphPad Prism version 8.0(GraphPad Software Inc., San Diego, CA, USA) were used for statistical analysis in the study.

## Results

A total of 80 patients from November 2018 to May 2020 were included in the final analysis of this study. During the process, 20 cases were excluded: 4 met the exclusion criteria, 6 refused to participate, 7 had open laparotomy, 2 had intraoperative bleeding > 2000 ml, and 1 had unclear ultrasound image (Fig. 1).

There were no significant differences in patient characteristics and operation data between the three groups, including age, weight, sex, education background, body mass index, ASA classification, duration of operation, duration of anesthesia, operation of liver segment, blood loss, length of incision for tissue removal, duration of hepatic porta block, time to anesthesia recovery, and time to extubation (Supplemental Table 1 and 2).

**Fig. 3:**
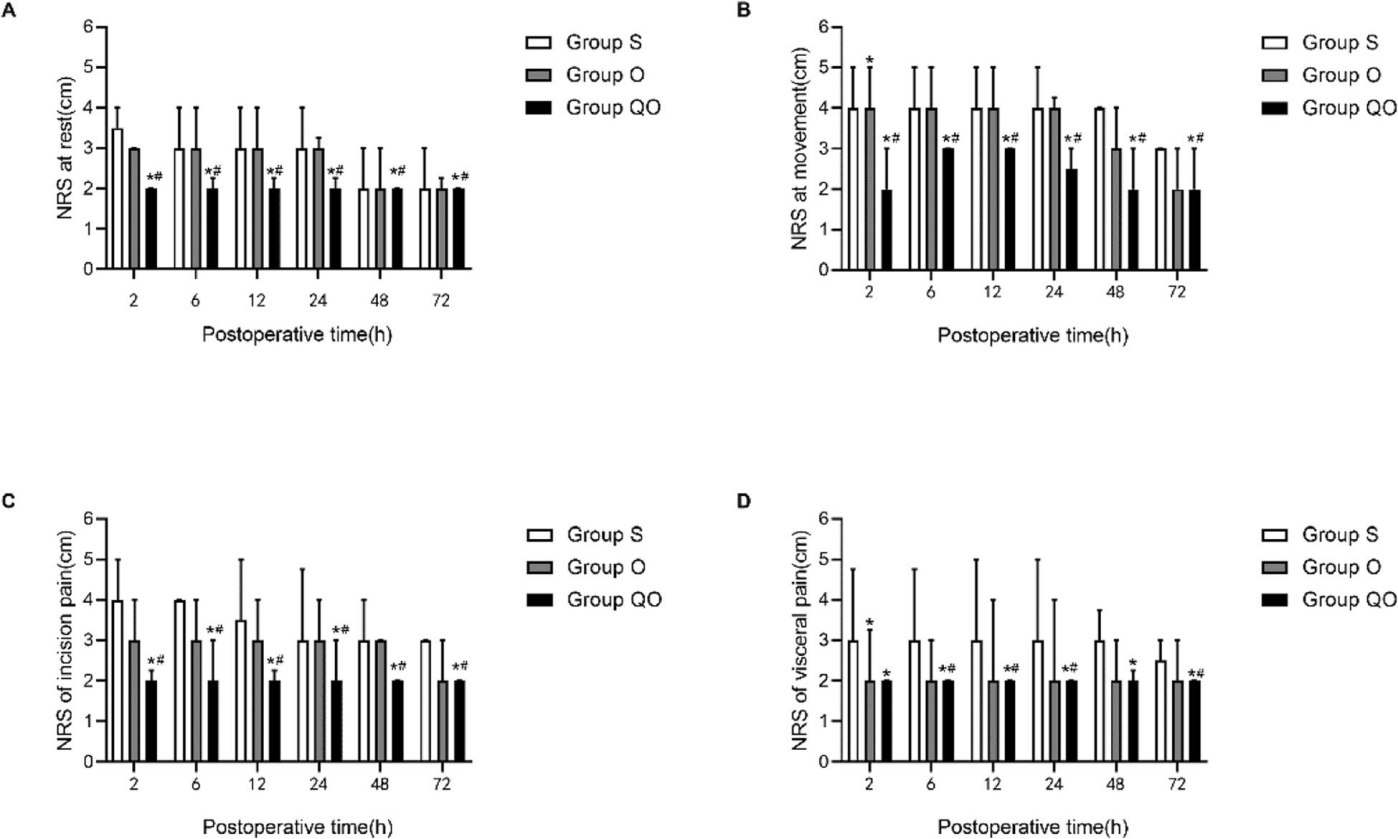
NRS scores after surgery. **a** NRS score at rest; **b** NRS score when coughing; **c** NRS score of incision pain; **d** NRS score of visceral pain. Data are shown as median (IQR). NRS score data were analyzed by ANOVA for repeated measures. *p < 0.05 compared with Group S;#*p* < 0.05 compared with Group O

In Group QO,the NRS score (when coughing) at 2, 6, 12, 24,48 and 72 h after surgery was lower than the Group S or Group O (*p* = 0.001) (Fig. 3b). In addition,the NRS scores(rest and incision pain) of 2, 6, 12, 24, 48 and 72 h in the Group QO were lower than those in the Group S and Group O (*p* < 0.05). There was no significant difference between group O and group S (Fig. 3a and c). Compared with the Group S, patients in the Group QO had lower visceral NRS pain score at 2, 6, 12, 24, 48, and 72 h after surgery (*p* < 0.05). And visceral NRS score of patients in Group O were lower than Group S at 2 h after surgery (*p* < 0.05). Compared with the Group O, patients in the Group QO had lower visceral NRS pain score at 2, 6, 12, 24 h after surgery (*p* < 0.05)(Fig. 3d).

**Fig. 4:**
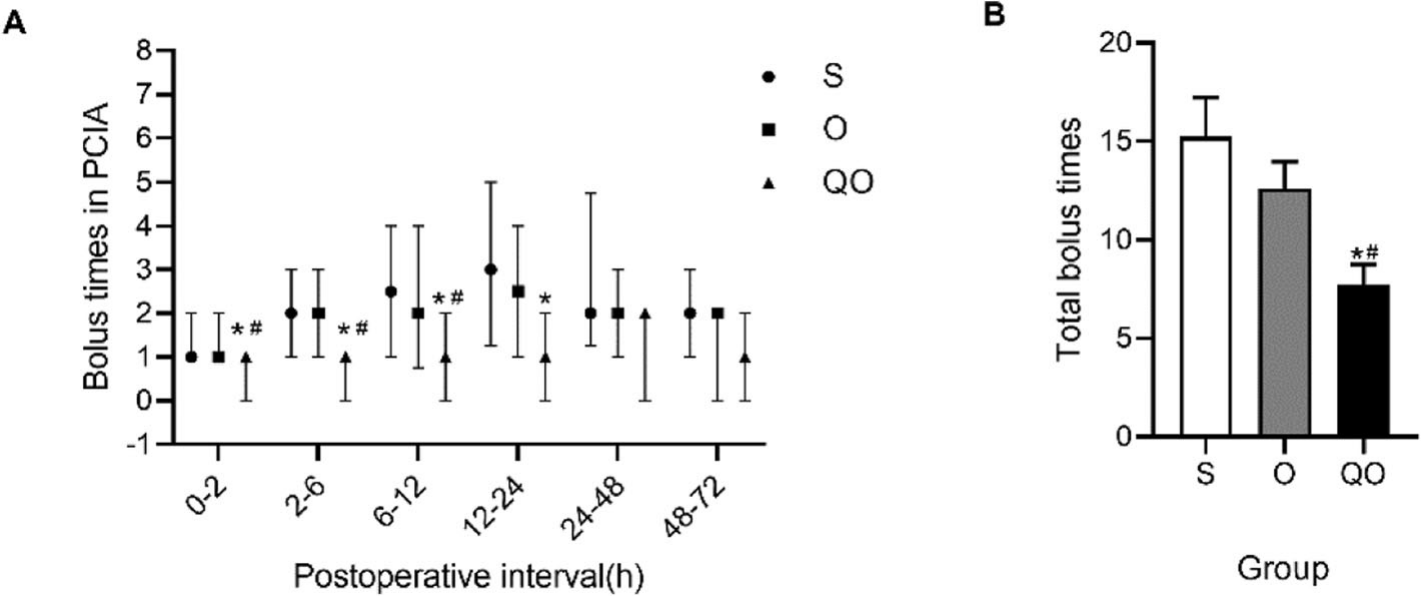
The comparison of visceral NRS pain score of three groups in different time point. **a** Bolus times in PCIA at postoperative interval; **b** Total bolus times. Data are shown as median (IQR). Data for Bolus times in PCIA at postoperative interval were analyzed by ANOVA for repeated measures. Data for total bolus times was analyzed by Kruskal-Wallis test for inter-group comparisons and Bonferroni method for pairwise comparison. *p < 0.05 compared with Group S;#*p* < 0.05 compared with Group O

Moreover, the number of patients in the Group QO who used PCIA independently in the PACU was less than the Group S and Group O(*p* < 0.05) and there was no statistical difference between Group O and Group S (Table 1). Within 24 h after surgery, the pressing times of PCIA in the QO group was reduced which was compared with the Group S and Group O(*p* < 0.05)(Fig. 4a). The total pressing times of PCIA in the Group QO were lower than the Group S and Group O(p < 0.05) (Fig. 4b);The equivalent dose of oxycodone consumed by the PCIA in Group QO was lower than the equivalent dose of sufentanil or oxycodone consumed by the PCIA in Group S and Group O(*p* < 0.05). The proportion of patients in the three groups who required additional analgesia was ranked as Group QO < Group O < Group S, and the difference between Group QO and Group S was statistically significant(*p* < 0.05) (Table 1).

**Table 1:** The use of PCIA

Secondary Outcomes		Group S (*n* = 20)	Group O (*n* = 30)	Group QO (*n* = 30)	*p*
**People of use bolus in PACU**	n(%)				**0.001**
Yes		14(82.4)	23(76.7)	9(30)^a,b^	
No		3(17.6)	7(23.3)	21(70)^a,b^	
**Opioid consumption in PCA after operation**	Median (IQR), ug or mg				
0-2nd h		3.0(2.1,5.0)	3.0(2.7,4.8)	2.7(1.0,3.0)^a,b^	0.051
2nd-6th h		6.0(4.0,8.0)	5.7(2.0,7.5)	3.6(2.0,4.1)^a,b^	**0.002**
6th–12th h		7.0(4.8,10.6)	6.8(3.8,10.8)	5.1(3.0,6.1)^a,b^	**0.007**
12th–24th h		12.0(8.5,15.5)	9.8(7.7,14.1)	9.4(6.0,11.4)	0.072
24th–48th h		16.0(14.0,21.5)	14.5(12.0,16.1)	14.0(12.0,15.4)^a^	**0.006**
48th–72th h		14.5(14.0,17.8)	15.1(12.0,15.6)	13.9(12.0,16.0)	0.134
Total		52.2(60.3,73.5)	53.5(48.5,70.0)	48.5(43.9,54.2)^a,b^	**0.002**
**Postoperative length of stay**	Median (IQR), d	8.0 (6.3,9.0)	7.0 (5.0,9.0)	7.0 (5.8,8.3)	0.376

Continuous variables were presented as median (IQR). Kruskal-Wallis test for inter-group comparisons and Bonferroni method for pairwise comparison. Qualitative variables were expressed as number of patients (percentage). The data were analyzed using Chi-squared test or Fisher’s exact test. ^a^:*p* < 0.05 compared with Group S; ^b^:*p* < 0.05 compared with Group O

*PCIA* Patient controlled intravenous analgesia

*PACU* Postanesthesia care unit

**Fig. 5:**
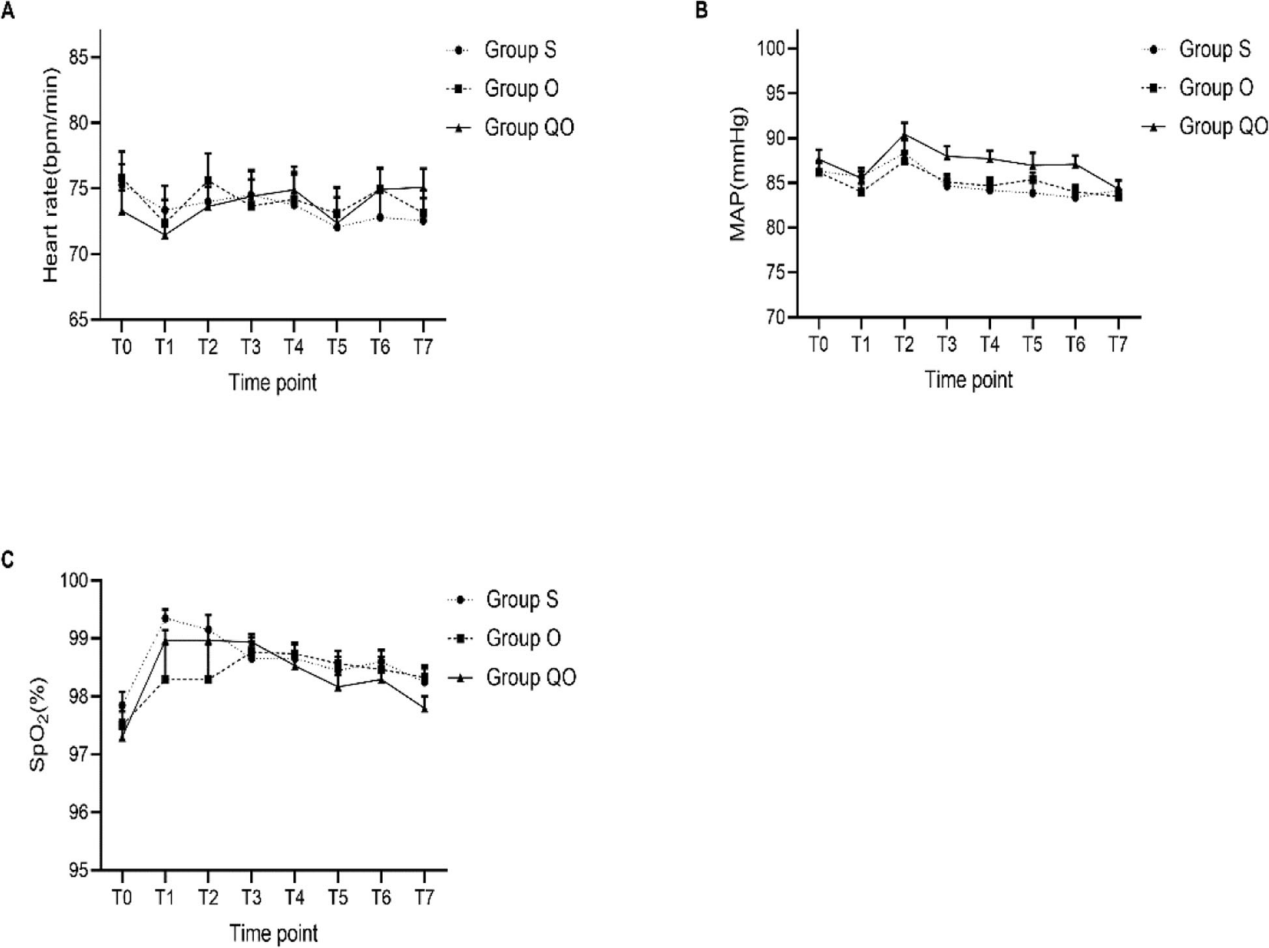
The comparison of vital signs of three groups in different time point. **a** Heart rate; **b** MAP, Mean Arterial Pressure; (C)SpO_2_, saturation of pulse oxygen; Data are shown as median (IQR). Data were analyzed by ANOVA for repeated measures *p < 0.05 compared with Group S; #*p* < 0.05 compared with Group O. Time point: T0:Before anesthesia; T1:Enter into PACU; T2:2h after operation; T3: 6 h after operation; T4:12h after operation; T5:24h after operation; T6:48h after operation; T7:72h after operation

However, there were no statistically significant differences in vital signs, Rammsay scores, postoperative vital signs, NRS nausea scores, NRS headache scores, incidence of flatulence time of first exhaust, time of first intaking liquid diet, time of first going to ground and time of removing the urine tube after the operation between the three groups (Fig. 5 and Supplemental Table 3).

The analgesic satisfaction of patients in Group QO was higher than the Group S (*p* = 0.001) and Group O (*p* = 0.012). There was no statistical difference in postoperative length of stay between the three groups (Supplemental Table 3).

We investigated the level of blockade in the 30 patients of Group QO at 6 h after operation and found that the levels were mainly at T8-T12(40%) (Supplemental Table 4).

## Discussion

In our study, the application of multimode analgesic regimen of TQLB combined with oxycodone-based PCIA could reduce postoperative NRS pain scores when coughing, total opioids consumption after laparoscopic hepatectomy and patients could have better analgesic satisfaction. However, the use of TQLB did not reduce nausea, time to mobilization, duration of hospital stays.

Over the recent years, quadratus lumborum block (QLB) has been increasingly use for postoperative analgesia following various types of surgical procedures such as cesarean, gynecological surgery, urinary, and orthopedic surgery [16–18]. Transmuscular quadratus lumborum block (TQLB) is one of the approaches in QLB. In our study, we found that TQLB performed immediately after surgery could significantly reduce NRS pain scores when coughing and oxycodone consumption in patients undergoing laparoscopic hepatectomy. Promil Kukreja [18] and colleagues used anterior quadratus lumborum block (QLB type 3) for postoperative analgesia after total hip arthroplasty, in which the duration of effective analgesia lasted for 48 h. Korgün Ökmen, et al. [19] applied posterior quadratus lumborum block (QLB type 2) in laparoscopic cholecystectomy and found that Visual Analogue Scale (VAS) score and the use of tramadol were significantly lower in the QLB group than those in the control group within 24 h after surgery. A recent meta-analysis also confirmed that single QLB significantly reduced the need for opioids during cesarean section and kidney surgery [20]. Based on the previous research, we chose TQLB as analgesic method after laparoscopic hepatectomy. Taken together, the above-mentioned results were in line with our findings.

Although the laparoscopic hepatectomy causes less tissue damage than open surgery, there are still significant incision pain, inflammatory pain, and visceral pain in the early postoperative stage. Of note, one strength of our study is to analyze pain after laparoscopic hepatectomy in four domains (pain at rest, pain when coughing, incision pain, visceral pain) according to the characteristics of the surgery. In our study, the NRS scores of all groups were averagely in mild intensity and the differences of total opioid consumption among the three groups were quite small which indicated that the postoperative analgesic methods of the three groups could meet the needs of analgesia after laparoscopic hepatectomy basically. However,both the NRS scores and opioid drug consumption in Group QO were minimum and the differences were statistically significant. Patients could benefit from the TQLB combined with oxycodone-based PCIA. By doing so, a comprehensive evaluation of the analgesic effects of TQLB was obtained. QLB has been applied in many clinical trials including this study, and in most these studies postoperative analgesia with PCIA is routinely used. The idea of continuous QLB has been introduced into practice recently. Qiang Zhu, et al. [21] conducted continuous QLB for postoperative analgesia in patients undergoing open liver resection. Their results showed that compared with the PCIA group, the continuous QLB group had significantly less postoperative cough and pain at 48 h after surgery, shortened time to mobilization and time to flatus, and acceleration in postoperative recovery. However, postoperative urinary retention may occur in patients receiving continuous QLB [22], and catheter displacement and tissue injury are also concerns. Therefore, cautions are needed to perform continuous QLB.

Compared with other techniques of abdominal wall blocks, one advantage of TQLB is that the location of the puncture needle and local anesthetic administration is less likely to reach the abdominal cavity, viscera, or large blood vessels and we choosed low concentration local anesthetic --30 ml of 0.25% ropivacaine which was injected into the interfascial plane on each side [23]. More importantly, circulatory and respiratory functions of the patients in our study remained stable throughout the study. No patient developed local anesthetic toxicity, lower limb paralysis, hematoma, or organ injury after TQLB.

Opioids play a major role in postoperative analgesia. A recent meta-analysis showed that patients who received postoperative patient-controlled analgesia (PCA) had better pain relief and satisfaction than patients who did not [24]. Oxycodone, a μ and κ receptor agonist, is widely used to control moderate to severe pain [25]. Several studies showed that oxycodone and morphine given in the equivalent dose by using the PCIA had comparable analgesic effects, while oxycodone produced a better visceral pain relief than morphine [6, 26]. Yi An and his colleagues found that compared with sufentanil, preoperative pain management with 0.1 mg/kg oxycodone could effectively inhibit visceral pain at 2 to 4 h after surgery, and reduce the level of inflammatory factors and serum tumor necrosis factor (TNF) [27]. Due to activity on κ receptors, oxycodone has been shown to better relieve visceral pain than morphine and fentanyl families. In our preliminary study, the patients reported very low visceral pain with oxycodone after laparoscopic hepatectomy. In this context, we selected oxycodone-based PCIA compared with sufentanil for postoperative pain control in this study. As a result, the visceral NRS pain scores of the patients in the QO group was significantly lower compared to the Group S and Group O at 2, 6, 12, and 24 h postoperatively, with a reduced total consumption of opioids for PCIA. And visceral NRS score of patients in Group O were lower than Group S at 2 h after surgery. One study showed that the local anesthetics could spread to the paravertebral space during TQLB, which could explain the effective visceral pain control of TQLB [28]. Actually,the thoracolumbar fascia (TLF),which is the anatomical basis of the QLB, is a layer of dorsal tissue extending from the thorax to the lumbar vertebra and surrounding the quadratus psoas, psoas major, and erector spinalis muscles. The thoracolumbar fascia has high density of sympathetic nerve fibers and abundant mechanical stimulation receptors [28, 29].

There are some limitations in our study. First, study is a single-blind study where the anesthesiology was aware of the group allocation due to the study design. Second, we did not we didn’t set group for TQLB compared with traditional opioids such as morphine or sufentanil. Finally, our study was a single center randomized controlled trial with a relatively small sample size, and thus selection bias may still exist. Well-designed multicenter randomized trials are needed to confirm our results.

## Conclusions

In Conclusion, TQLB combined with oxycodone-based PCIA provided satisfactory postoperative analgesia and reduced oxycodone consumption in patients following laparoscopic hepatectomy. Moreover, compared with sufentanil-based PCIA,oxycodone-based PCIA provided better analgesia of visceral pain.

## Supplementary Information

**Additional file 1: Table S1**. Patient Characteristics.

**Additional file 2: Table S2**. Operation data.

**Additional file 3: Table S3**. Postoperative recovery.

**Additional file 4: Table S4**. Block plane for sensitive loss at 6 h after operation.

## Data Availability

The datasets used and/or analysed during the current study are available from the corresponding author on reasonable request.

## Abbreviations

TQLB: Transmuscular quadratus lumborum block
PCIA: patient-controlled intravenous analgesia
Group S: Sufentanil for PCIA group
Group O: Oxycodone for PCIA group
Group QO: Transmuscular quadratus lumborum block + oxycodone for PCIA group
NRS: Numerical Rating Scale
IQR: interquartile range
HCC: Hepatocellular carcinoma
ERAS: Enhanced recovery after surgery
ASA: American Society of Anesthesiologists
QL: Quadratus lumborum
