# Supplementary material for: Ultrasound-guided transmuscular quadratus lumborum block reduced postoperative opioids consumptions in patients after laparoscopic hepatectomy: a three-arm randomized controlled trial
Source: BMC Anesthesiol. 2021 Feb 11;21:45. doi: 10.1186/s12871-021-01255-3 (PMC7877010; doi:10.1186/s12871-021-01255-3)
Supplement: Supplementary file 1 — Additional file 1: Table S1. Patient Characteristics. [file 12871_2021_1255_MOESM1_ESM.doc]

**Supplemental table 1. Patient Characteristics**

| Characteristic |  | Group S  (n=20) | Group O  (n=30) | Group QO  (n=30) | *p* |
| --- | --- | --- | --- | --- | --- |
| **Age** | Median  (IQR)，  years of age | 53.0  (44.5,62.0) | 48  (43.7,52.1) | 46  (42.5,50.2) | 0.060 |
| **Sex** | n(%) |  |  |  | 0.493 |
| Male |  | 12(60.0) | 22(73.3) | 23(76.6) |  |
| Female |  | 8(40.0) | 8(26.7) | 7(23.4) |  |
| **Body mass index** | Median  (IQR),  kg/m^2^ | 22.3  (19.5,23.7) | 22.8  (21.5,23.5) | 23.4  (21.3,23.5) | 0.596 |
| **Education background** | n(%) |  |  |  | 0.534 |
| Primary school or below |  | 6(30.0) | 3(10.0) | 5(16.6) |  |
| Junior high school |  | 6(30.0) | 11(36.6) | 6(20.0) |  |
| Higher school |  | 6(30.0) | 10(33.4) | 12(40.0) |  |
| Bachelor degree  or above |  | 2(10.0) | 6(20.0) | 7(23.2) |  |
| **ASA** | n(%) |  |  |  | 0.524 |
| Ⅰ |  | 1(5) | 2(6.6) | 3(10) |  |
| Ⅱ |  | 6(80.0) | 27(90.0) | 26(86.6) |  |
| Ⅲ |  | 3(15.0) | 1(3.3) | 1(3.3) |  |

a:*p*<0.05 compared with Group S; b:*p*<0.05 compared with Group O.

Continuous variables were presented as median (IQR). Kruskal-Wallis test for inter-group comparisons and Bonferroni method for pairwise comparison. Qualitative variables were expressed as number of patients (percentage). The data were analyzed using Fisher’s exact test.
