# Supplementary material for: Ultrasound-guided transmuscular quadratus lumborum block reduced postoperative opioids consumptions in patients after laparoscopic hepatectomy: a three-arm randomized controlled trial
Source: BMC Anesthesiol. 2021 Feb 11;21:45. doi: 10.1186/s12871-021-01255-3 (PMC7877010; doi:10.1186/s12871-021-01255-3)
Supplement: Supplementary file 2 — Additional file 2: Table S2. Operation data. [file 12871_2021_1255_MOESM2_ESM.doc]

**Supplemental table 2. Operation data**

| Characteristic |  | Group S  （n=20） | Group O  （n=30） | Group QO  （n=30） | *p* |
| --- | --- | --- | --- | --- | --- |
| **Surgery of liver segment** | n(%) |  |  |  | 0.193 |
| left lateral lobe of liver |  | 7(35.0) | 6(20) | 11(36.7) |  |
| S2 |  | 0(0.0) | 2(6.7) | 0(0.0) |  |
| S3 |  | 0(0.0) | 0(0.0) | 1(3.3) |  |
| S4 |  | 0(0.0) | 2(6.7) | 1(3.3) |  |
| S5 |  | 0(0.0) | 5(16.7) | 3(10.0) |  |
| S6 |  | 2(10.0) | 8(26.6) | 3(10.0) |  |
| S7 |  | 2(10.0) | 1(3.3) | 0(0.0) |  |
| S8 |  | 5(25.0) | 3(10.0) | 5(16.7) |  |
| Left half liver |  | 3(15.0) | 3(10.0) | 3(10.0) |  |
| Right half liver |  | 1(5.0) | 3(10.0) | 3(10.0) |  |
| **Duration of surgery** | Median  (IQR),min | 197.5  (142.5,330.0) | 195.0  (176.4,246.5) | 194.0  (160.5,221.2) | 0.343 |
| **Duration of anesthesia** | Median  (IQR),min | 315.0  (246.3,410.0) | 277.5  (265.4,336.5) | 286.0  (245.3,314.4) | 0.222 |
| **Blood loss** | Median  (IQR),ml | 200.0  (100.0,375.0) | 200  (183.3,287.3) | 200.0  (168.7,325.9) | 0.546 |
| **Incision of removing the specimen** | n(%) |  |  |  | 0.089 |
| Yes |  | 14(70.0) | 28(93.3) | 24(80.0) |  |
| No |  | 6(30.0) | 2(6.7) | 6(20.0) |  |
| **Blocking hepatic porta** |  |  |  |  | 0.567 |
| Yes |  | 6(30.0) | 12(40.0) | 8(26.6) |  |
| No |  | 14(70.0) | 18(60.0) | 22(73.3) |  |
| **Time on blocking hepatic porta** | Median  (IQR),min | 0.0  (0.0,12.5) | 0.0  (0.0,17.5) | 0.0  (0.0,6.3) | 0.224 |
| **Time of anesthesia recovery after the operation** | Median  (IQR),min | 45.0  (40.0,58.0) | 45.0  (40.7,46.4) | 45.0  (43.1,47.3) | 0.352 |
| **Time of tracheal tube extraction after the operation** | median(IQR),min | 56.0  (45.0,64.8) | 55.0  (50.0,56.0) | 52.0  (51.5,54.9) | 0.880 |

a:*p*<0.05 compared with Group S; b:*p*<0.05 compared with Group O.

Continuous variables were presented as median (IQR). Kruskal-Wallis test for inter-group comparisons and Bonferroni method for pairwise comparison. Qualitative variables were expressed as number of patients (percentage). The data were analyzed using Chi-squared test or Fisher’s exact test.
