# Supplementary material for: Ultrasound-guided transmuscular quadratus lumborum block reduced postoperative opioids consumptions in patients after laparoscopic hepatectomy: a three-arm randomized controlled trial
Source: BMC Anesthesiol. 2021 Feb 11;21:45. doi: 10.1186/s12871-021-01255-3 (PMC7877010; doi:10.1186/s12871-021-01255-3)
Supplement: Supplementary file 3 — Additional file 3: Table S3. Postoperative recovery. [file 12871_2021_1255_MOESM3_ESM.doc]

**Supplemental table 3. Postoperative recovery**

| Postoperative recovery |  | Group S  （n=20） | Group O  （n=30） | Group QO  （n=30） | *p* |
| --- | --- | --- | --- | --- | --- |
| **Nausea and vomiting** | n(%) | 12(60.0) | 15(50.0) | 13(43.3) | 0.537 |
| **dizziness** | n(%) | 9(45.0) | 15(50.0) | 13(43.3) | 0.884 |
| **Ramsay**  **Score** | median(IQR) |  |  |  |  |
| 2h |  | 2.0(2.0,3.0) | 2.0(2.0,2.0) | 2.0(1.9,2.0) | 0.092 |
| 6h |  | 2.0(2.0,2.0) | 2.0(2.0,2.0) | 2.0(2.0,2.0) | 0.789 |
| 12h |  | 2.0(2.0,2.0) | 2.0(2.0,2.0) | 2.0(2.0,2.0) | 0.572 |
| 24h |  | 2.0(2.0,2.0) | 2.0(2.0,2.0) | 2.0(2.0,2.0) | 0.900 |
| 48h |  | 2.0(2.0,2.0) | 2.0(2.0,2.0) | 2.0(2.0,2.0) | 0.900 |
| 72h |  | 2.0(2.0,2.0) | 2.0(2.0,2.0) | 2.0(2.0,2.0) | 0.900 |
| **Bowelsound** | median(IQR)，times |  |  |  |  |
| 2h |  | 0.0(0.0,1.0) | 1.0(0.0,1.0) | 1.0(0.0,1.0) | 0.401 |
| 6h |  | 1.0(0.0,1.0) | 2.0(1.0,2.3) | 1.0(0.0,2.0) | 0.062 |
| 24h |  | 2.0(1.3,3.0) | 2.5(2.0,3.5) | 3.0(2.0,3.0) | 0.201 |
| 48h |  | 3.0(2.0,3.8) | 2.0(2.0,4.0) | 3.0(2.0,4.0) | 0.568 |
| 72h |  | 4.0(3.0,4.0) | 3.0(3.0,4.0) | 3.0(3.0,4.0) | 0.603 |
| **Time of first**  **exhaust** | median(IQR),  h | 42.0(30.5,49.5) | 44.0(30.8,61.3) | 43.0(39.3,61.0) | 0.554 |
| **Time of first intaking** | median(IQR),  h | 37.5(30.0,40.8) | 40.5(30.8,47.3) | 40.0(24.0,47.3) | 0.655 |
| **Time of first** **going to ground** | median(IQR),  h | 41.0(37.6,48.5) | 41.0(25.8,55.5) | 44.5(29.8,55.3) | 0.444 |
| **Time of** **removing the urine tube** | median(IQR),  h | 41.0(28.0,47.5) | 41.0(25.0,48.0) | 44.0(26.0,54.3) | 0.754 |
| **Analgesic satisfaction** | median(IQR) | 4.0(3.0,4.0) | 4.0(4.0,4.0)^a^ | 4.5(4.0,5.0)^a,b^ | **0.001** |

Continuous variables were presented as median (IQR). Kruskal-Wallis test for inter-group comparisons and Bonferroni method for pairwise comparison. Qualitative variables were expressed as number of patients (percentage). The data were analyzed using Chi-squared test or Fisher’s exact test. a:*p*<0.05 compared with Group S; b:*p*<0.05 compared with Group O.
