# Supplementary material for: Ultrasound-guided transmuscular quadratus lumborum block reduced postoperative opioids consumptions in patients after laparoscopic hepatectomy: a three-arm randomized controlled trial
Source: BMC Anesthesiol. 2021 Feb 11;21:45. doi: 10.1186/s12871-021-01255-3 (PMC7877010; doi:10.1186/s12871-021-01255-3)
Supplement: Supplementary file 4 — Additional file 4: Table S4. Block plane for sensitive loss at 6 h after operation. [file 12871_2021_1255_MOESM4_ESM.doc]

**Supplemental table 4** **Block plane for sensitive loss at 6 hours after operation**

| Block plane for sensitive loss at 6 hours after operation | n(%) |
| --- | --- |
| T7-T12 | 9(30) |
| T8-T12 | 12(40) |
| T8-L1 | 2(7) |
| T10-T12 | 3(10) |
| T10-L1 | 4(13) |
